# Changes over Time in Intracranial Air in Patients with Cerebral Air Embolism: Radiological Study in Two Cases

**DOI:** 10.1155/2015/491017

**Published:** 2015-11-12

**Authors:** Yoko Kaichi, Shingo Kakeda, Yukunori Korogi, Tomohisa Nezu, Shiro Aoki, Masayasu Matsumoto, Makoto Iida, Kazuo Awai

**Affiliations:** ^1^Diagnostic Radiology, Graduate School and Institute of Biomedical and Health Sciences, Hiroshima University, Hiroshima, Kasumi 1-2-3, Minami-ku, Hiroshima 734-8551, Japan; ^2^Department of Radiology, University of Occupational and Environmental Health School of Medicine, Iseigaoka 1-1, Yahatanishi-ku, Kitakyushu 807-8555, Japan; ^3^Department of Clinical Neuroscience and Therapeutics, Hiroshima University Graduate School of Biomedical and Health Sciences, Hiroshima, Kasumi 1-2-3, Minami-ku, Hiroshima 734-8551, Japan

## Abstract

Cerebral air embolism can be easily identified on computed tomography (CT) scans. However, changes in the distribution and amount of intracranial air are not well known. We report two patients with cerebral air embolism and present imaging findings on the serial changes in the intracranial air. We thought that the embolic source was venous in one patient because CT showed air inflow in cortical veins in the bilateral frontal areas, reflecting air buoyancy. In the other patient, CT showed air inflow into not only the cortical veins but also the bilateral cerebral hemispheres and we thought this to be a paradoxical cerebral air embolism. We found that intracranial air can be promptly absorbed and while cerebral infarcts due to air are clearly visualized on diffusion-weighted images (DWI), the air may rapidly disappear from images. In patients with suspected cerebral air embolism whose CT findings show no intracranial air, DWI should be performed because it may reveal cerebral infarction due to cerebral air embolism.

## 1. Introduction

Cerebral air embolism (CAE) is a well-known complication of trauma, central venous catheterization, pressurized intravenous infusion systems, and orthopedic, neurosurgical, and cardiovascular surgical procedures [1]. Some CAEs can be identified on brain computed tomography (CT) scans and subsequent infarcts on diffusion-weighted images (DWI) [2, 3]. However, changes in the distribution and amount of intracranial air have not been demonstrated in detail. We report two patients with cerebral air embolism and discuss the serial changes in their intracranial air.

## 2. Case Reports

### 2.1. Patient 1

**Figure 1:**
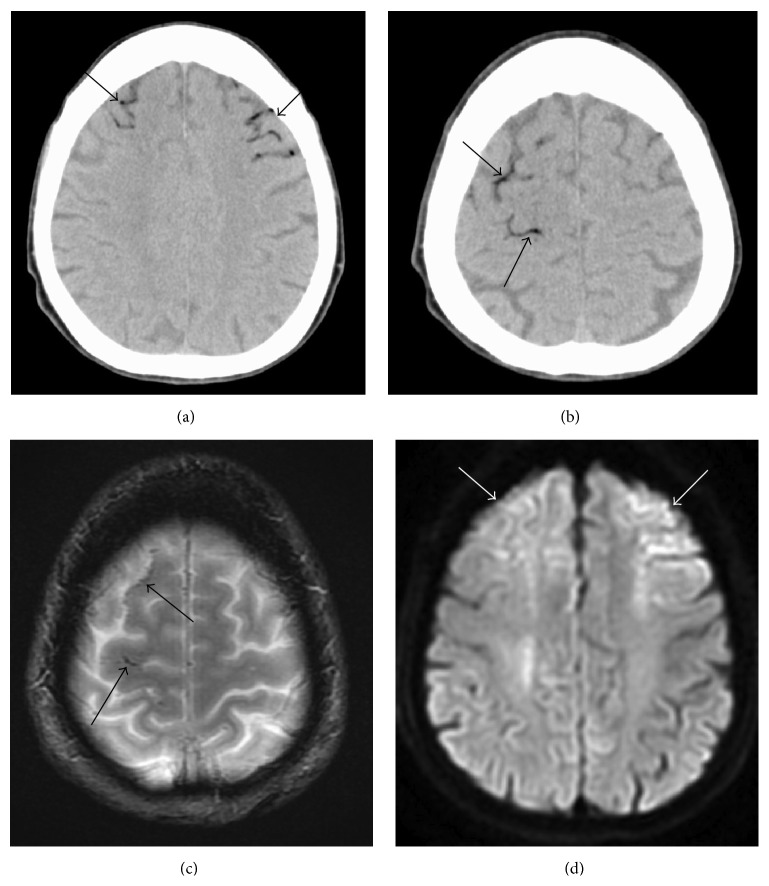
A 70-year-old woman (case 1). (a, b) Brain CT performed immediately after her arrival at our hospital (60 min after the estimated CAE onset) shows air inflow in cortical veins in the bilateral frontal areas and the right parietal area reflecting buoyancy of the air (arrows). (c) T2^*∗*^WI obtained 30 minutes after the CT scan at the same level as in (b). There was less air than in (b) (arrows). (d) DWI obtained 30 min after the CT scan showing cortical areas with restricted diffusion near the air (arrows).

**Figure 2:**
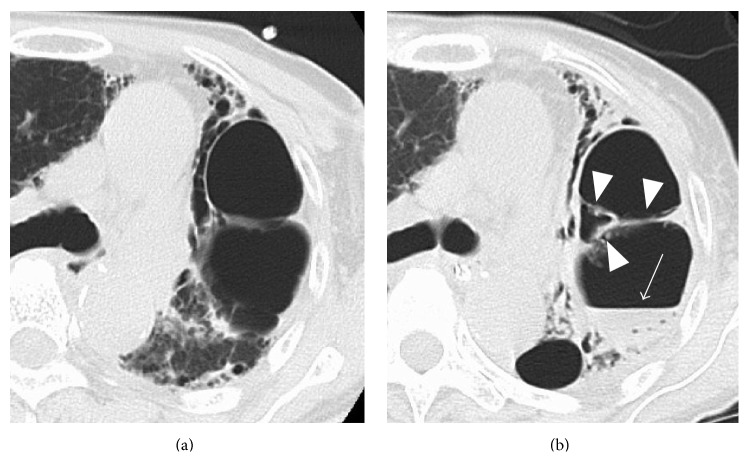
A 70-year-old woman (case 1). (a) Chest CT performed immediately after her arrival at our hospital (60 min after the estimated CAE onset). There is a giant bulla (8 cm in diameter) with septa in the left upper lobe. (b) Chest CT scan obtained 10 days after her admission shows collapse of the bulla with fluid collection (arrow) and thickening of the septa (arrow heads).

A 70-year-old woman with interstitial pneumonia suddenly lost consciousness. She was taken to a local hospital by ambulance. Emergent brain CT and magnetic resonance imaging (MRI) scans were normal and one hour later she walked to her car. However, 15 min thereafter her consciousness declined again. She arrived at our hospital by ambulance 5 hr after the initial symptom onset, 45 min after her consciousness decreased for the second time. Neurological examination showed left hemiparesis and she was comatose. Her body temperature was 38.2°C and she was coughing. Blood test revealed a white blood cell count of 10.80 × 10^3^/*μ*L and C-reactive protein of 1.89 mg/dL. Emergent brain CT images revealed air inflow in the cortical veins in the bilateral frontal and parietal areas (Figures 1(a) and 1(b)). MRI scans were performed 30 min later. T2^*∗*^-weighted images showed tortuous air inflow in the bilateral frontal areas; it was less than what had been observed on the CT images (Figure 1(c)). DWI revealed the cortical areas with restricted diffusion near the air (Figure 1(d)). On a chest CT scan there was air in the right ventricle and the left external jugular vein and there was a giant bulla (8 cm in diameter) with septa in the left upper lobe (Figure 2(a)). A diagnosis of CAE was made and she was treated with edaravone. Hyperbaric oxygen therapy could not be delivered because she was too unstable for transfer to a facility with a hyperbaric chamber.

She gradually regained consciousness and the air had disappeared on brain CT scans obtained 12 hr after her admission. Six days after her admission the high intensity area on DWI scans had spread and become clear.* Aspergillus flavus* was examined with sputum culture and serous antibodies to it were determined. A chest CT scan obtained 10 days after her admission showed collapse of the bulla with fluid collection and thickening of the septa (Figure 2(b)). Under the hypothesis that the alveolar changes were due to aspergillosis, she was treated with voriconazole and the infection abated. A transthoracic echocardiogram was normal. Although her consciousness level improved gradually and she was able to speak, her left arm and leg remained hemiplegic. She was transferred to a rehabilitation hospital two months later.

### 2.2. Patient 2

**Figure 3:**
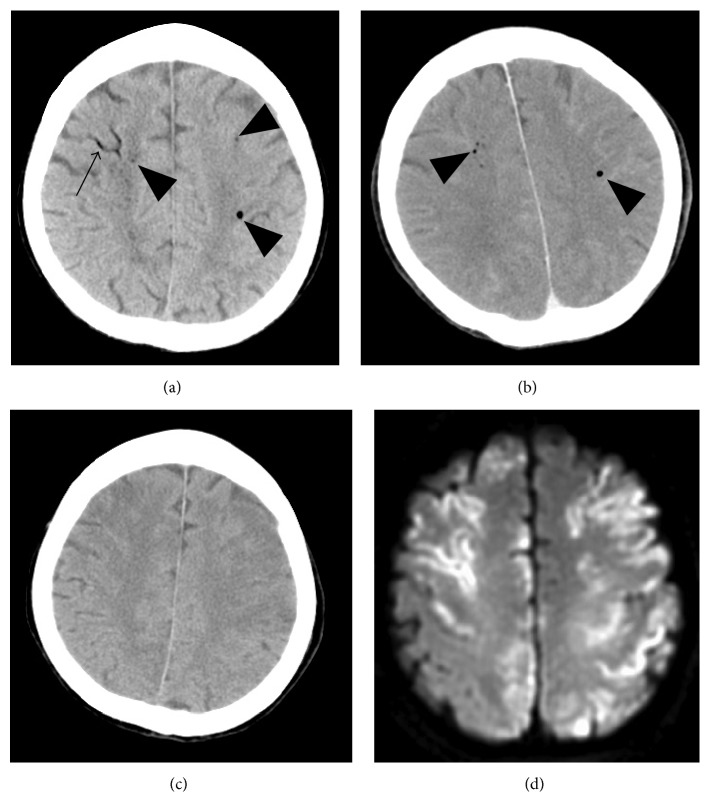
An 82-year-old woman (case 2). (a) Brain CT performed 30 min after notice of her consciousness decrease, that is, 60 min after the estimated onset of CAE, shows air inflow in the cortical veins (arrow) and bilateral cerebral hemispheres (arrow heads). (b) Brain CT obtained 50 min after the first CT scan. The air densities (arrow heads) were diminished. (c) Brain CT obtained 18 hr after the first CT scan. The air densities had completely disappeared. (d) DWI performed one week after onset showing multiple areas of restricted diffusion affecting primarily the cortical areas in the bilateral hemispheres adjacent to air bubbles.

This 82-year-old woman with myasthenia gravis was seen at a local hospital complaining of dyspnea. Two days later her dyspnea worsened (PaO2 76.3 mmHg, PaCO2 99.1 mmHg). She underwent noninvasive positive-pressure ventilation; however, it was unable to correct dyspnea. Therefore, she subsequently underwent tracheal intubation and placement of a central venous catheter in the right internal jugular vein. Although her blood gas analysis improved (PaO2 143.0 mmHg, PaCO2 32.7 mmHg) she remained unable to breathe on her own. The development of a myasthenic crisis was suspected and she was admitted to our hospital the next day. Steroid pulse therapy and an immunoadsorbent technique were performed. Her respiratory condition was stable on the 9th hospital day and she was extubated. On the 10th hospital day the attending physician who had come to exchange the central venous catheter noticed a decline in her consciousness. She had been seen to brush her teeth 30 min earlier but the onset of the consciousness decline was uncertain. An emergent brain CT scan (Figure 3(a)), obtained 30 min after her consciousness decline was noted, revealed air inflow in the cortical veins, the bilateral cerebral hemispheres, the bilateral cavernous sinuses, and the sella turcica, consistent with the anterior intercavernous venous sinus. A chest CT scan showed air in the left brachial vein. CAE was diagnosed and she was treated with oxygen inhalation and intravenous injections of edaravone. Hyperbaric oxygen therapy could not be delivered because she showed a deterioration of consciousness and was too unstable for transfer to a facility with a hyperbaric chamber. The air density was markedly diminished on a brain CT scan obtained 50 min later (Figure 3(b)) and not observed on brain CT scans acquired 18 hr after the first scan (Figure 3(c)). MRI scans were performed a week later. DWI showed multiple areas of restricted diffusion affecting predominantly the cortical areas in the bilateral hemispheres adjacent to air, the corpus callosum, and the cerebellum (Figure 3(d)). A transesophageal echocardiogram (TEE) with injection of agitated saline was normal. Her consciousness level improved gradually although she manifested paralysis of the left leg and both arms. She was transferred to a rehabilitation hospital 3 months later.

## 3. Discussion

Intravascular air can lead to both arterial and venous infarcts. Venous cerebral air emboli may result from retrograde movement of air into the jugular veins, particularly if the patient is sitting upright. Venous air can become arterial if the volume of the air embolus exceeds the capacity of the pulmonary filter [4] or if there is right-to-left intracardiac shunting (paradoxical CAE). Mechanisms leading to air embolism ischemia are blood flow obstruction, vasospasm, and thrombus formation due to platelet activation.

Some previous studies have indicated that the blood brain barrier may be broken by air bubbles migrating on to arteries and arterioles due to damage of endothelium [5, 6]. Furthermore, the air bubbles cause not only air embolism resulting from mechanical obstruction leading to ischemia but also inflammatory reactions such as margination and activation of leukocytes resulting in a cerebral edema or a secondary ischemia [7].

The preferential location of venous air emboli in the frontal cortical area may be related to the position of the patient (sitting or supine) at the time of air entry. This hypothesis is supported by findings in a CAE patient who experienced cerebellar and occipital infarcts in the prone position [8]. Cerebral infarcts are often observed in cortical areas near air [2, 9]. The distribution of the intracranial air in one of our cases (patient 1) agrees with this hypothesis. We think that her CAE derived from a venous source and it may have been attributable to the passage of air into the bronchial veins through a bronchovenous fistula and/or damaged pulmonary vessels because she manifested a giant bulla in the left lung and infection. We speculate that her coughing ruptured the bulla of the lung, which caused air accumulation within the right ventricle resulting in outflow obstruction and her unconsciousness. Her biphasic unconsciousness also indicates CAE [10]. Serpiginous air densities, a finding compatible with venous CAE [9], were observed along the frontal and parietal cortical sulci. In addition, DWI showed cortical areas with restricted diffusion, a finding made in the acute phase of cerebral infarction, near the air. The wide spreading of the hyperintensity at follow-up DWI might be caused by the secondary inflammatory reactions due to the air bubbles.

In patient 2, DWI showed multiple sites of restricted diffusion affecting predominantly cortical areas not only near the air but also in widespread regions of the bilateral hemispheres. Some air may have already disappeared when the first CT scan was acquired. Others [2, 9] detected intracranial air on CT scans performed less than 7 hours after the onset of CAE. While Jeon et al. [2] reported that brain CT performed 38 hours after the onset of CAE still showed multiple punctate air densities in the left frontal subcortices, Suzuki et al. [3] detected no air density on brain CT scans acquired 30 minutes after the onset of CAE considered as arterial. When dogs were injected with 0.5 or 2 mL of air directly into the carotid artery, 50% and 100% of the animals, respectively, manifested arterial CAE; the half-life of air absorption was approximately 8 minutes [11]. Inoue et al. [9] reported a patient with arterial CAE who harbored a small amount of air in the cerebral parenchyma on brain CT scans performed immediately after onset. They suggested that the rapid injection of a large amount of air could elicit arterial CAE. The duration of air visible on CT or MRI scans may depend on the amount of air infusion and whether the CAE is venous or arterial.

Some brain CT scans of arterial or paradoxical CAE revealed punctate air densities in the brain parenchyma [9]. Because the surface tension of air bubbles is inversely correlated with their diameter, smaller air bubbles are more resistant to rupture than larger bubbles. Air bubbles are more likely entrapped in small end arteries in the cortical layers and brain parenchyma than in larger proximal arteries [12]. With respect to the pattern of infarction of arterial or paradoxical CAE, multiple brain infarctions and infarcts in frontal and parietal areas have been documented [13].

In one of our cases (patient 2), a brain CT scan obtained an hour after the estimated CAE onset revealed intracranial air including the brain parenchyma. DWI showed multiple infarcts not only in frontal and parietal but also in occipital areas, suggestive of paradoxical CAE. Although her TEE with the injection of agitated saline was normal, we cannot exclude the presence of an undetectable small intracardiac shunt such as a patent foramen ovale. Moreover, she had undergone noninvasive positive-pressure ventilation. While the lungs normally act as effective filters allowing only bubbles smaller than 22 *μ*m to transverse the pulmonary vascular bed, their filtering capacity may be compromised by pulmonary barotrauma from positive-pressure ventilation [14].

The treatment of venous CAE consists of the immediate termination of any central line procedures in progress. The patient should be placed in the Trendelenburg position and rotated towards the left lateral decubitus position to trap air in the apex of the ventricle and to prevent its ejection into the pulmonary arterial system or its movement retrogradely into the cerebral venous circulation. Hyperbaric oxygen therapy is also recommended [15] because its high pressure reduces the volume of the air embolus and hyperoxygenation facilitates gas removal via denitrogenation, thereby maintaining oxygenation in the ischemic tissues. Even CT is normal and there is CAE suspected, hyperbaric oxygen therapy is seriously to be considered as soon as possible [16].

CAE originating from a venous source often goes unrecognized because of its nonspecific symptoms, generalized seizure, unconsciousness, dizziness, chest pain, nausea, visual disturbances and headache [17, 18], but should be suspected in patients with inexplicable neurologic findings in the presence of a venous rout or respiratory disease. Such patients should undergo a brain CT scan as soon as possible because a prompt diagnosis and adequate treatment avoid further cerebral damage. If CAE is suspected despite the absence of intracranial air on CT scans, DWI should be performed because it may reveal cerebral infarction observed mainly in cortical areas coincident with edema due to CAE, although the air was quickly absorbed.
